# Machine Learning Driven Window Blinds Inspired Porous Carbon‐Based Flake for Ultra‐Broadband Electromagnetic Wave Absorption

**DOI:** 10.1002/advs.202521130

**Published:** 2026-01-13

**Authors:** Zhe Wang, Wanchong Li, Lu Feng, Zaiqing Yang, Shicheng Jin, Dongxu Zhao, Xiaoyong Wu, Yan Wang, Yu Mao, Jinsong Zhang

**Affiliations:** ^1^ Institute of Structured and Architected Materials Liaoning Academy of Materials Shenyang China; ^2^ College of Materials Science and Engineering Shenyang University of Chemical Technology Shenyang China; ^3^ College of Physical Science and Technology Shenyang Normal University Shenyang China; ^4^ Aviation Key Laboratory of Science and Technology on Electromagnetic Environment Effects Shenyang China; ^5^ National Key Laboratory of Electromagnetic Information Control and Effects Shenyang China

**Keywords:** electromagnetic wave absorption, localization, machine learning, magneto‐electric coupling, porous carbon

## Abstract

The core challenge in developing lightweight and high‐efficiency electromagnetic wave absorbing materials lies in achieving a decoupled improvement in impedance matching and loss performance while reducing thickness. Inspired by the structure of window blinds, this study proposes and designs a Discrete Slat Tunable Electromagnetic Wave Absorption Material (DSTEAM). By incorporating a magneto‐electric coupling concept and an artificial intelligence‐assisted data‐driven optimization strategy, the successfully fabricated DSTEAM exhibits outstanding performance with a reflection loss below –10 dB over an ultra‐broadband frequency range of 2.6–40 GHz, while maintaining a thin thickness of only 9.85 mm and an areal density of 0.566 kg/m^2^. The superior performance of DSTEAM is attributed to gradient‐induced multiple scattering at discrete sheet interfaces, a synergistic enhancement of localized field strength, and a magneto‐electric coupling modulation mechanism. This AI‐driven collaborative design strategy offers a novel concept and effective pathway for the development of next‐generation lightweight and broadband electromagnetic wave absorption materials.

## Graphical abstract

Inspired by the regulation mechanism of window blinds, this study designs an electromagnetic wave‐absorbing metamaterial. By introducing the magneto‐electric coupling concept and integrating it with an artificial intelligence‐based data‐driven collaborative optimization strategy, the material optimizes impedance matching performance and enhances loss capability while reducing its thickness. Within the ultra‐broad frequency range of 2.6–40 GHz, this material achieves excellent performance with a reflection loss below –10 dB.

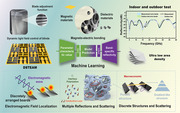

## 1 Introduction

The iterative evolution of information and communication technologies is accelerating the development of an intelligent, hyper‐connected society. Wireless communication systems, represented by 5G/6G, are not only driving societal digitalization but also giving rise to complex electromagnetic radiation environments [1, 2]. According to the International Telecommunication Union (ITU), the number of global electronic devices has exceeded 30 billion. The broad‐bandwidth, high‐intensity electromagnetic waves generated by these devices not only interfere with the signal integrity of military radars and satellite navigation systems but may also pose biological safety risks to humans. [3, 4, 5] Against this backdrop, the development of novel electromagnetic wave absorption materials (EWAMs) characterized by broad bandwidth, strong absorption, thin thickness, and light weight has become a common technical imperative to address electromagnetic pollution [6, 7, 8].

EWAMs must satisfy two core principles: First, the material must exhibit wave impedance($Z=\sqrt{\mu /\varepsilon }\approx 377\Omega$)similar to that of free space to minimize interfacial reflection and facilitate efficient penetration of electromagnetic waves into the material, thereby introducing magneto‐electric coupling. Second, through multi‐physical field coupling effects such as interfacial polarization, hysteresis loss, and multiple scattering, the incident electromagnetic energy should be efficiently converted into thermal energy for dissipation. However, high‐loss characteristics often coincide with impedance mismatch, leading to increased reflection. This physical antagonism between high loss and weak matching severely constrains breakthroughs in EWAM performance [9, 10].

To enhance the performance of EWAMs, researchers have primarily pursued two strategies: tuning material composition and designing structural topology. In terms of material composition, concepts such as magneto‐electric coupling have been introduced to balance dielectric and magnetic losses. For instance, Lei et al. [11] incorporated FeCo@C to promote interfacial polarization loss and enhance the synergy between dielectric and magnetic losses, achieving a minimum reflection loss of −61.04 dB and an effective absorption bandwidth (EAB) of 7.25 GHz. In structural design, porous topology optimization has proven effective. For example, Zhong et al. [12] constructed a three‐dimensional porous structure, attaining a minimum reflection loss value of −63.39 dB at 10.06 GHz and an EAB of 4.81 GHz (7.49–12.30 GHz). Although porous materials have significantly improved wave absorption performance, there remains room for enhancement in terms of lightweight and efficient absorption. Sun et al. [13] proposed a sharp conical structural design that achieved an EAB of 37.6 GHz (2.4–40 GHz) at a thickness of 18 mm. While such tapered designs can improve impedance matching, their acute geometric features reduce the effective volume ratio, leading to a new contradiction between structural lightweighting and strong decay in loss performance. To compensate for this loss, researchers often increase material thickness. For example, Yang et al. [14] designed a pyramid structure that extended the EAB to 2–40 GHz at a thickness of 40 mm. Although these structural optimization strategies have yielded positive results, such thickness‐compensation approaches face implementation challenges in space‐sensitive applications [15]. Therefore, a key scientific challenge in the field of EWAMs is how to simultaneously optimize impedance matching without increasing material thickness or sacrificing loss capability.

Bionic design provides abundant inspiration for material structural innovation, while machine learning offers a powerful tool for high‐performance optimization. The integration of these two approaches opens up novel pathways for the design of electromagnetic wave‐absorbing materials [16, 17, 18, 19, 20, 21, 22]. Inspired by the structure of window blinds and incorporating a magneto‐electric coupling design concept, this study developed a Discrete Slat Tunable Electromagnetic Absorber (DSTEAM). A neural network was employed to establish a predictive model for absorption performance, while a genetic algorithm was applied to achieve intelligent multi‐parameter optimization. Furthermore, Smith charts and electromagnetic field distribution analyses were utilized to elucidate the mechanism through which the structural design enhances absorption performance. DSTEAM is expected to overcome the thickness increase typically associated with enhancing impedance matching and loss performance, while also providing new insights for the design of broadband, lightweight electromagnetic absorbing materials.

## 2 Results and Discussion

### 2.1 Structural Characteristics

#### 2.1.1 Structural Design

**FIGURE 1:**
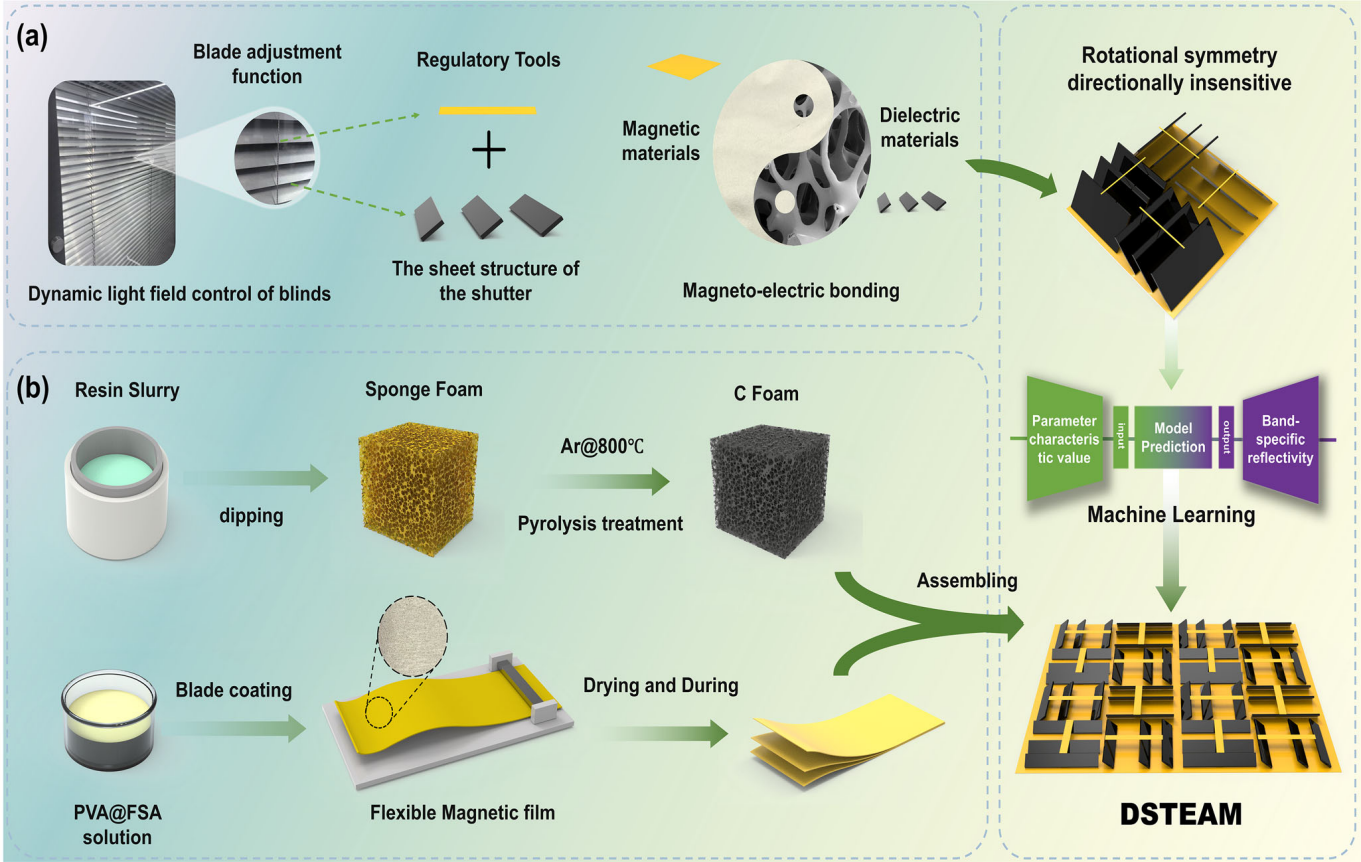
(a) Inspiration source and magneto‐electric coupling design concept of the discrete slat tunable electromagnetic wave absorption material inspired by the structure of window blinds. (b) Fabrication process of the porous carbon material and the FeSiAl magnetic material.

Window blinds, through specific arrangement methods, can block excessive light under varying illumination conditions while promoting air circulation. Inspired by the dynamic light‐field modulation mechanism of blinds, this study proposes a DSTEAM. As illustrated in Figure 1a, its core structure consists of rotationally symmetric arranged porous carbon sheet units, a bottom iron‐silicon‐aluminum (FeSiAl) magnetic substrate, and a top magnetic strip tuning component, forming a dielectric–magnetic–structural triple synergistic regulation system.

Based on the principle of dynamic gradient regulation of the light field by window blinds, mechanical adjustment of the slat inclination angle (analogous to the opening and closing of blind slats) enables dynamic optimization of the spatial gradient distribution of the equivalent permittivity and permeability. This approach breaks through the limitations of conventional absorbing materials, which are often restricted to a fixed structure with a narrowband response. The porous carbon material was prepared using a template replication method combined with pyrolysis, and the magnetic material was fabricated via doctor blade coating (Figure 1b). Furthermore, machine learning was employed to optimize the structural parameters and explore the optimal configuration for specific electromagnetic responses. This design extends the mechanical tunability of window blinds into the realm of electromagnetic regulation, offering a new paradigm for broadband reconfigurable absorption materials. It shows promising potential for applications in intelligent stealth structures and adaptive electromagnetic absorption.

#### 2.1.2 Characterization of Porous Carbon and Magnetic Materials

**FIGURE 2:**
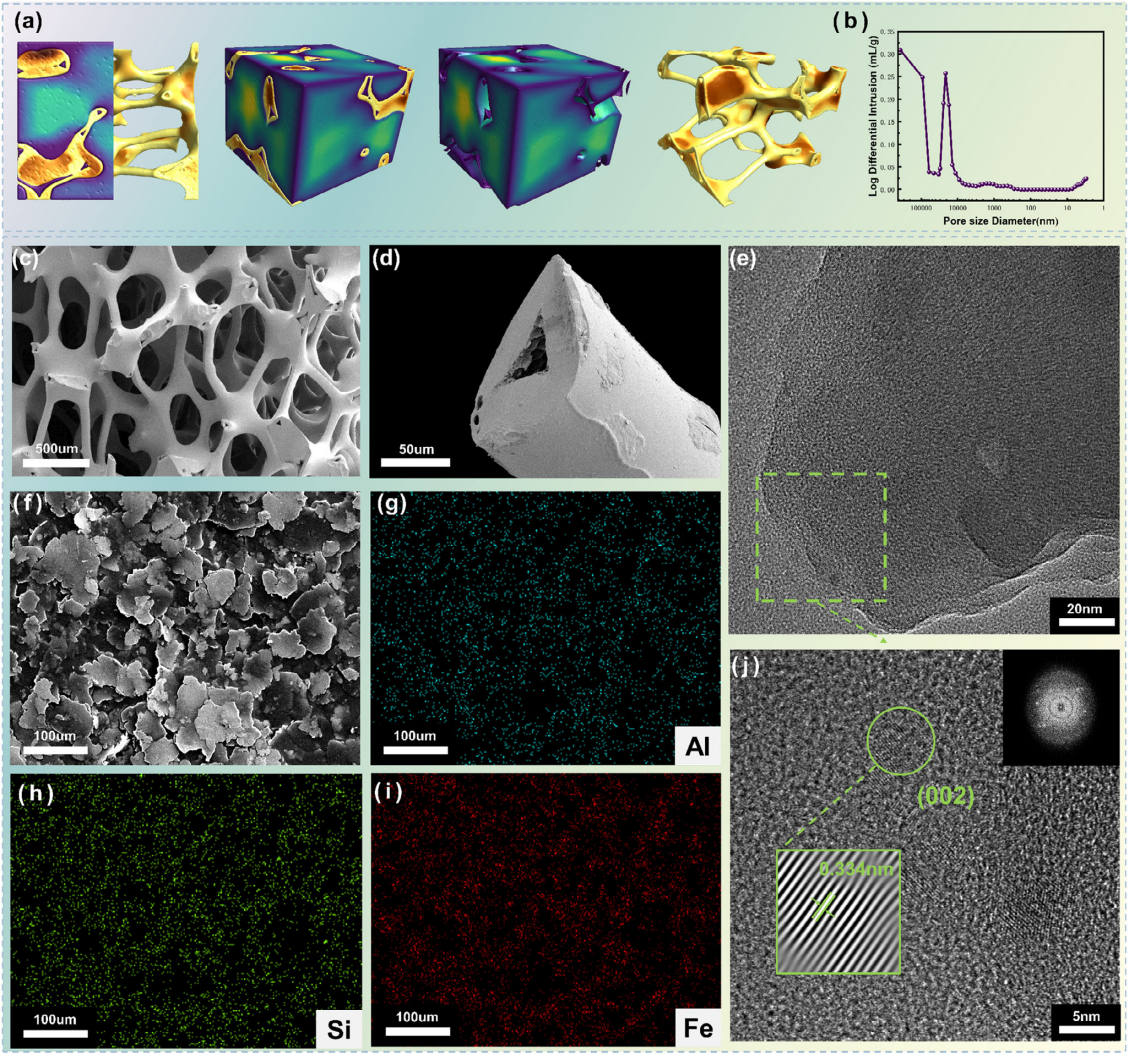
(a) 3D‐XRT model. (b) Mercury intrusion porosimetry (MIP) test of the porous carbon. (c) Microscopic morphology of the porous carbon. (d) SEM image showing the microscopic morphology of a single porous carbon skeleton. (f) SEM image of FeSiAl powder and the corresponding EDS mappings for (g) aluminum, (h) silicon, and (i) iron. (e, j) TEM images of the porous carbon.

To elucidate the micro‐morphology and structural characteristics of the material, high‐resolution X‐ray micro‐computed tomography (3D‐XRT) was first employed to reconstruct the in‐situ pore network within the material (Figure 2a), providing an authentic three‐dimensional geometric model for subsequent electromagnetic simulations. Three‐dimensional pore statistical analysis yielded a porosity of 87.566%. Concurrently, the total porosity of the porous carbon material measured by the water displacement method was 87.5%. These results are in close agreement. Mercury intrusion porosimetry (MIP) indicated that triangular pores within the carbon skeleton accounted for 5.1% of the total porosity, with the pore diameter primarily concentrated around 20 µm (Figure 2b). Scanning electron microscopy (SEM) observations revealed that the porous carbon sample exhibits a rich three‐dimensional pore structure, with pore sizes mainly distributed within the range of 200–400 µm (Figure 2c). Notably, the skeleton diameter of the porous carbon is approximately 80 µm, and the carbon skeleton itself exhibits a hollow structure (Figure 2d). This unique hierarchical pore architecture (macro‐pores + hollow skeleton) provides ample space for multiple reflections and scattering of electromagnetic waves [23]. SEM observation of the FeSiAl powder (Figure 2f) showed its typical flaky morphology. Combined with energy‐dispersive X‐ray spectroscopy (EDS) elemental mapping (Figure 2g–i), a uniform distribution of Fe, Si, and Al elements was clearly detected. The weight percentages were approximately Al: 7.7%, Si: 11.4%, and Fe: 81.0%.

**FIGURE 3:**
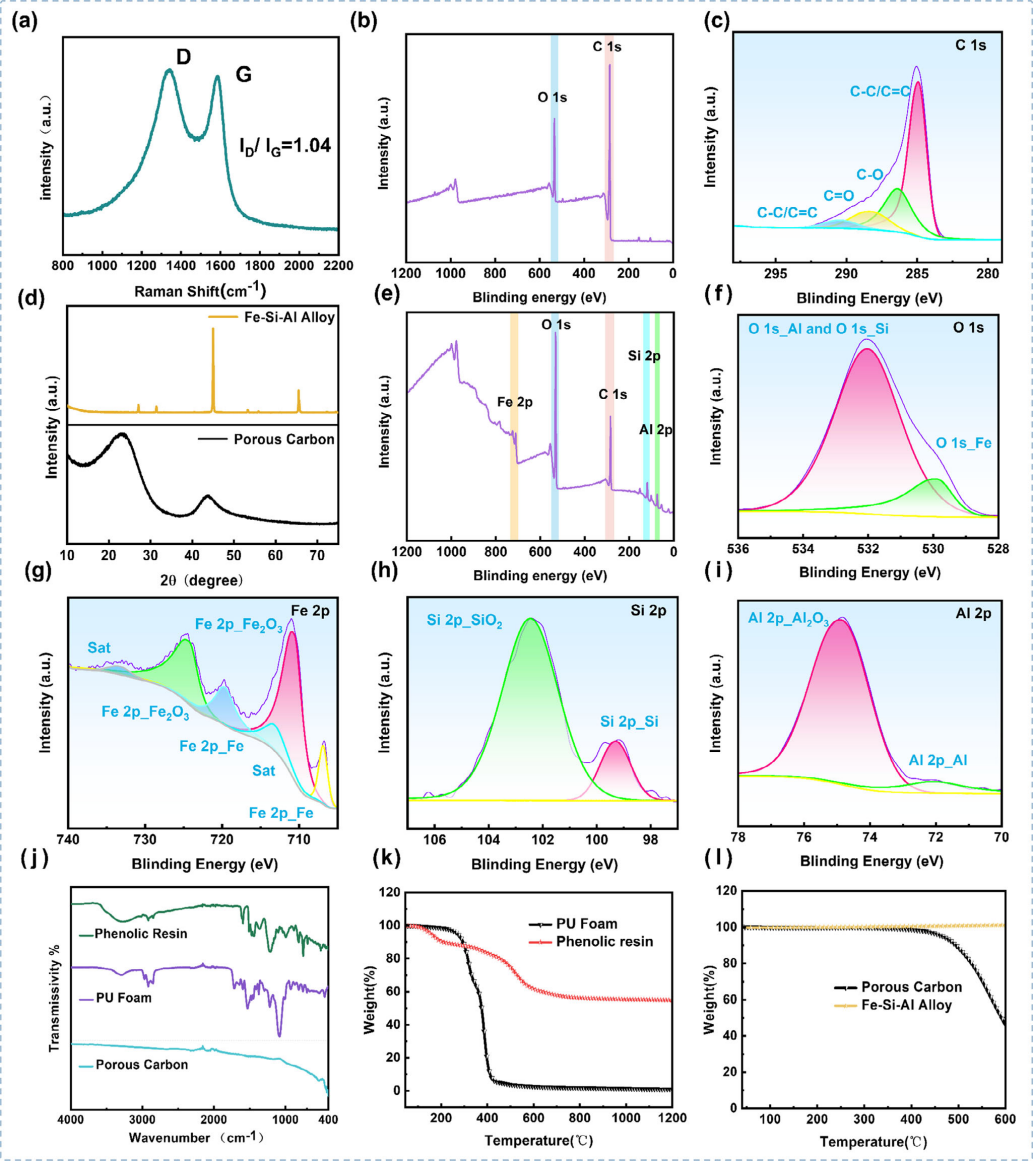
(a) Raman spectrum of the porous carbon. (b) XPS survey spectrum and (c) high‐resolution C 1s spectrum of the porous carbon. (d) X‐ray diffraction (XRD) patterns of the FeSiAl alloy and the porous carbon. (e) XPS survey spectrum of FeSiAl, along with its corresponding high‐resolution (f) O 1s, (g) Fe 2p, (h) Si 2p, and (i) Al 2p spectra. (j) Fourier transform infrared (FT‐IR) spectra of the precursors (PU foam and phenolic resin) and the porous carbon. (k) Thermogravimetric (TG) curves of the precursors under an argon atmosphere. (l) TG curves of the porous carbon and the FeSiAl alloy under an air atmosphere.

Transmission electron microscopy (TEM) images (Figure 2e) further reveal the local fine structure of the porous carbon. Dispersed dark regions are visible, exhibiting clear lattice fringes (Figure 2j), indicating the presence of short‐range ordered microcrystalline domains. The measured characteristic lattice spacing is 0.334 nm, which closely matches the standard interplanar spacing of the graphite (002) crystal plane [24]. To evaluate the overall crystalline state, X‐ray diffraction (XRD) analysis was performed (Figure 3d). The sample exhibits broad diffraction peaks at approximately 24° and 44°, corresponding to the (002) and (100) crystal planes of graphitic carbon, respectively. This broadening feature clearly indicates that the material is predominantly amorphous carbon [25], which is consistent with the TEM observation of localized small graphitic microcrystals with (002) orientation (i.e., globally disordered but locally ordered). Raman spectroscopy (Figure 3a) provides quantitative information on the graphitization degree and defects in the carbon material. The spectrum of the porous carbon shows distinct characteristic peaks at 1353 cm^−^^1^ (D band, originating from disorder/defects) and 1600 cm^−^^1^ (G band, attributed to in‐plane vibrations of sp^2^ carbon). The intensity ratio ID/IG = 1.04 is significantly greater than 1, further confirming the low graphitization degree and high defect density of the sample [26, 27], which is highly consistent with the strong amorphous characteristics indicated by XRD. The XRD pattern of the FeSiAl (Figure 3d) shows sharp diffraction peaks near 44.6° and 65.0°, which are assigned to the (110) and (200) crystal planes of the FeSiAl, respectively. These characteristic peaks unambiguously confirm that the sample consists of a crystalline FeSiAl.

The chemical composition and elemental valence states of porous carbon and FeSiAl were further analyzed using X‐ray photoelectron spectroscopy (XPS). The XPS survey spectrum of the porous carbon (Figure 3b) exhibited two characteristic peaks at 284.8 and 532.16 eV, corresponding to C 1s and O 1s, respectively. This indicates that the carbon skeleton and some oxygen‐containing functional groups were retained in the precursors (PU foam and phenolic resin) after pyrolysis. The high‐resolution C 1s spectrum (Figure 3c) was deconvoluted into four peaks, assigned to C─C/C═C (≈284.51 eV), C─O (≈285.47 eV), C═O (≈286.96 eV), and O─C═O (≈290.31 eV) [28]. The Fourier transform infrared (FT‐IR) spectra of the precursors and the porous carbon are shown in Figure 3j, confirming the presence of oxygen‐containing functional groups in the precursors. The thermogravimetric (TG) curves of the precursors under an argon atmosphere (Figure 3k) indicate a certain degree of mass loss persisting beyond 800°C. Combined with the XPS analysis of the porous carbon, this suggests that a small portion of the oxygen‐containing functional groups in the precursors was not completely decomposed during pyrolysis. The oxygen‐containing structures in the porous carbon are thus related to the pyrolysis residues of the phenolic resin and PU foam.

The XPS survey spectrum of the FeSiAl material shows it contains elements such as O, Fe, Si, and Al (Figure 3e). Deconvolution of the high‐resolution Fe 2p region (Figure 3g) reveals that iron exists in mixed valence states, including Fe^3^^+^ and metallic Fe^0^. The characteristic peaks of Fe^0^ (707 and 720 eV) confirm the presence of a small amount of metallic iron in the material. However, its surface has been partially oxidized, forming an oxide layer dominated by Fe^3^^+^ [29, 30]. Integrating the high‐resolution Fe 2p, Si 2p, and Al 2p spectra (Figure 3g–i), it is concluded that the material surface is primarily composed of Fe^3^^+^, Al^3^^+^, and Si^4^^+^ (corresponding to Fe_2_O_3_, Al_2_O_3_, SiO_2_), with minor contributions from metallic Fe^0^, Al^0^, and Si^0^. The XPS analysis, along with the XRD characterization of the FeSiAl bulk phase (Figure 3d), indicates that the bulk phase is an Fe‐Si‐Al alloy, while surface elements have undergone partial oxidation upon environmental exposure [31, 32].

To evaluate the thermal stability of the materials, thermogravimetric analysis was performed on both the porous carbon and FeSiAl in an air atmosphere. The results are shown in Figure 3l. The TG curve of the porous carbon shows that its mass retention rate remained above 95% throughout heating from room temperature to 450°C, demonstrating excellent thermal stability. The FeSiAl material exhibited a minimal mass increase in the high‐temperature region of the test. This slight mass gain is attributed to the reaction with oxygen during heating, forming a thin oxide layer, while also confirming its good thermal stability within this temperature range [33].

**FIGURE 4:**
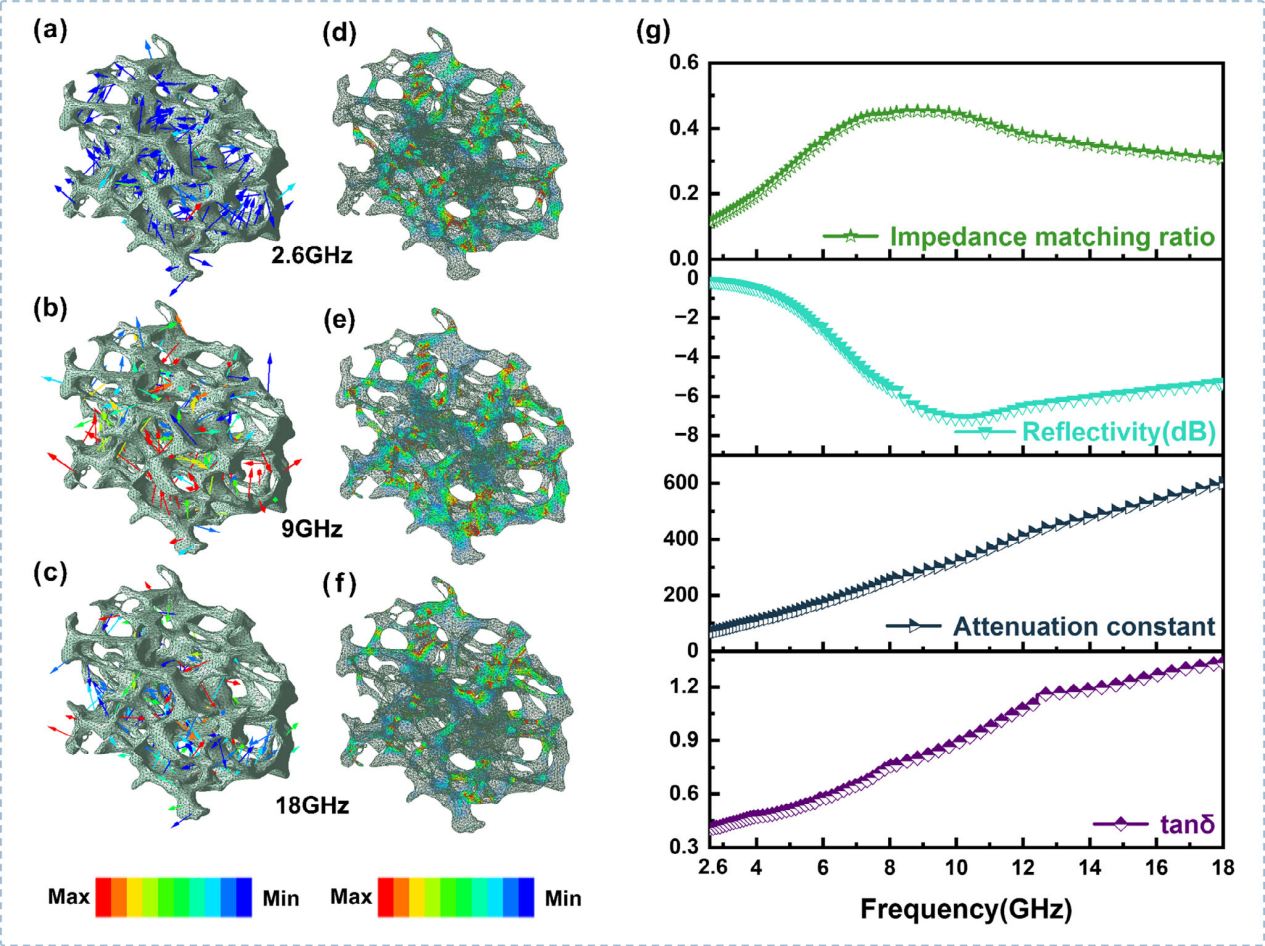
(a–c) In situ simulation analysis of the Poynting vector field for the 3D‐XRT model of the porous carbon material at 2.6, 9, and 18 GHz, respectively. (d–f) In situ simulation analysis of the energy loss field for the 3D‐XRT model of the porous carbon material at 2.6, 9, and 18 GHz, respectively. (g) Impedance matching curve, reflection coefficient (or reflectivity) curve, attenuation constant curve, and dielectric loss tangent curve for the porous carbon.

To elucidate the structure–property relationship between the poreskeleton architecture of porous carbon materials and their electromagnetic waveabsorbing performance, this study integrates 3DXRT technology with finiteelement electromagnetic field simulations. This approach enables quantitative characterization of the in situ pore structure and coupled analysis of the electromagnetic performance. Based on the realistic 3DXRT structural model, in situ simulations of the Poynting vector field at different frequencies were performed using the HighFrequency Structure Simulator (HFSS). The results clearly demonstrate (Figure 4a–c) that at 2.6, 9, and 18 GHz, electromagnetic waves undergo multiplescattering effects within the complex pore network of the porous carbon. This effectively extends the multiplereflection paths, increases the interaction time between the waves and the material, and enhances the loss capability of the material. Among these frequencies, the vector field intensity is strongest at 9 GHz. Further analysis of the energy dissipation field (Figure 4d–f) confirms this trend: the porouscarbon skeleton exhibits pronounced loss characteristics at all frequencies, with the strongest energy distribution occurring at 9 GHz, consistent with the Poynting vector field distribution. This reveals the critical regulatory role of the material's structure on its waveabsorption performance.

The relationship between the reflectivity of the porous carbon and its impedance matching, as well as attenuation coefficient was further calculated (Figure 4g). The results show that among the three studied frequencies—2.6, 9, and 18 GHz—the best impedance matching occurs at 9 GHz, leading to stronger wavepenetration capability and the lowest reflectivity. The loss capability of the material increases with frequency, reaching its maximum at 18 GHz, where the impedance matching is relatively poorer. The superior impedance matching at 9 GHz results in stronger interaction between the electromagnetic waves and the material, which is consistent with the 3DXRT simulation results.

#### 2.1.3 Influence of Discrete Slat Structural Parameters on the Wave‐Absorbing Performance of the Material

**FIGURE 5:**
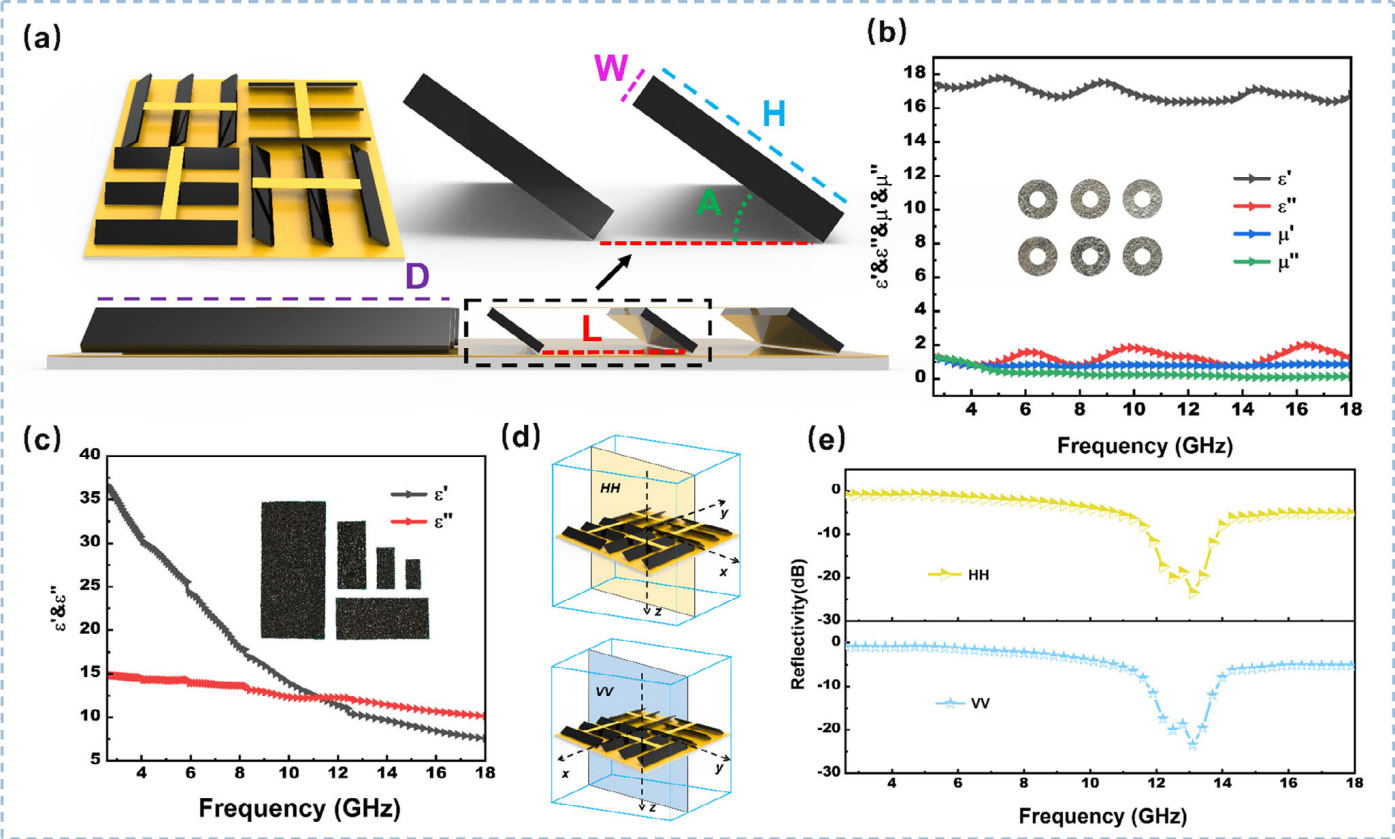
(a) Definition of DSTEAM structural parameters. (b) Complex permittivity and complex permeability of FeSiAl. (c) Complex permittivity of porous carbon. (d) Schematic diagram of HH and VV polarization directions. (e) Comparison of DSTEAM reflectivity under HH and VV polarization directions.

In the design of broadband, lightweight electromagnetic wave‐absorbing materials, the precise control of structural parameters is a central challenge in balancing impedance matching and loss performance. As illustrated in Figure 5a, this study systematically defines four key parameters based on the rotationally symmetric design of the DSTEAM, tilt angle (A), carbon sheet width (W), carbon sheet height (H), and inter‐slit distance (L). The electromagnetic parameters of the materials serve as essential inputs for finite element simulations and provide the foundation for multi‐physics coupled analysis. The experimentally measured electromagnetic parameters of the FeSiAl magnetic material and the porous carbon material within the frequency range of 2.6–18 GHz are presented in Figure 5b,c, respectively.

In practical applications of electromagnetic wave absorbing materials, it is essential to consider not only a single polarization direction but also both vertical polarization (HH) and horizontal polarization (VV). As illustrated in Figure 5e, the reflectivity under both polarization modes was evaluated for the structural configuration with A = 50°, H = 3 mm, W = 2 mm, and L = 54 mm. Owing to the rotational symmetry of DSTEAM, no significant difference in reflectivity was observed between the two polarization directions. Therefore, only the HH polarization direction was selected for the subsequent parameter sweep study in this work.

**FIGURE 6:**
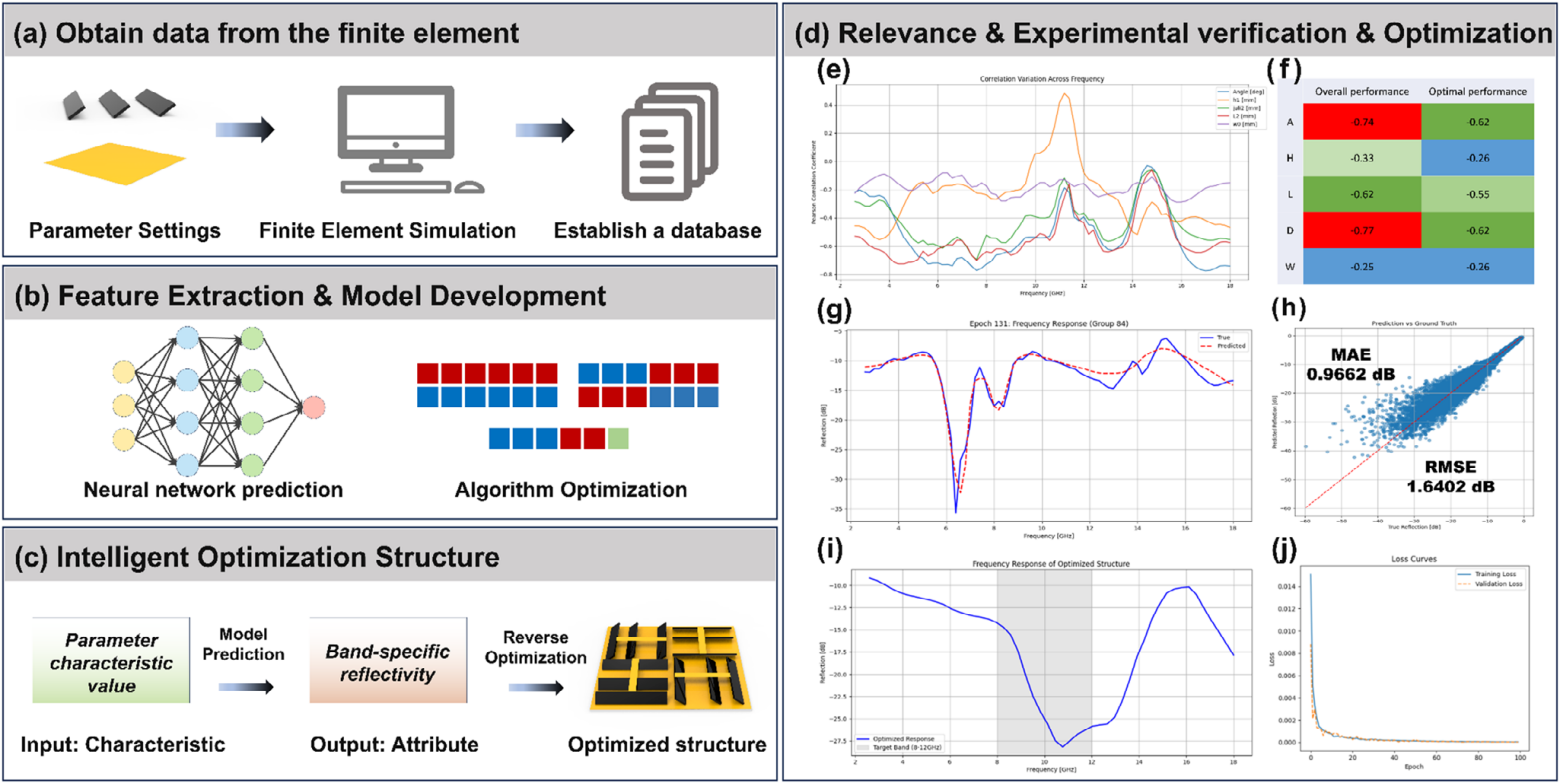
(a) Data acquisition process; (b) Algorithm development and training; (c) Evaluation, prediction, and optimization; (d) Correlation analysis and experimental validation & optimization: (e and f) Parameter correlation analysis, (g) Prediction vs. test set comparison, (h) Coefficient of determination and its error, (i) Full‐band and specific‐band optimization, (j) Loss value.

The microwave absorption performance of the material primarily depends on the intrinsic electromagnetic wave absorption efficiency of the porous carbon material and magnetic material, structural parameters, and the frequency of the electromagnetic wave. As shown in Figure 6, varying combinations of the structural parameters (A, W, H, and L) of the DSTEAM material in this work significantly influence its absorption performance. Parameter correlation analysis (Figure 6e,f) indicates that the relationship between each geometric parameter and the structural reflectivity performance is not a simple linear mapping or point‐to‐point correlation; instead, performance modulation arises from complex nonlinear interactions and synergistic effects among multiple parameters. The ultimate impact of altering any single parameter strongly depends on the current state of other parameters, suggesting that the structure is a highly coupled system that requires holistic and synergistic optimization. However, different structural parameters combine in such a way that even a small number of varying parameters can generate an exponential number of combinations. This vast array of geometric configurations makes the search for optimal structural parameters highly challenging. A potential solution to overcome the challenge of exploring possible geometric configurations of the material is to employ machine learning‐driven approaches, such as inverse design and reinforcement learning, to efficiently navigate the complex structural design space [34, 35]. This study adopts a data‐driven design approach integrating finite element simulation, fully connected neural networks, and genetic algorithms to predict and optimize tunable structures targeting specific structural parameters. The objective is to efficiently explore optimized structures for specific electromagnetic responses, thereby enabling the rapid selection of superior structural designs.

After 200 epochs of iterative training, the neural network‐based surrogate model demonstrated exceptional predictive accuracy, with a coefficient of determination (R^2^) = 0.97, mean absolute error (MAE) = 1.6402 dB, and root mean square error (RMSE) = 0.9662 dB. Driven by a genetic algorithm, its inverse design strategy enables broadband absorption and optimization for specific frequency bands. Compared to finite element method (FEM) simulations, the surrogate model improves prediction speed by four orders of magnitude—achieving a single electromagnetic response prediction in just 12 ms (vs. 360 s for FEM)—while maintaining a field distribution deviation rate ≤0.3%. As shown in Figure 6, this intelligent optimization framework, through a closed‐loop process of “parameter generation–surrogate prediction–objective evaluation,” compresses the design cycle to 1/50th of that required by traditional trial‐and‐error methods, providing an efficient paradigm for the rapid inverse design of electromagnetic metamaterials.

### 2.2 Optimization and Fabrication

**FIGURE 7:**
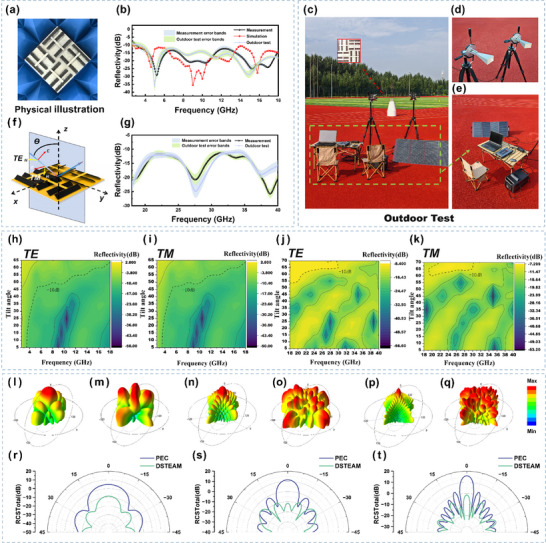
(a) Photograph of the DSTEAM material. (b) Comparison between measured and simulated results (including error bands) for DSTEAM in the 2.6–18 GHz range. (c) Fieldtesting setup. (d) Horn antenna for the 18–40 GHz range. (e) Fieldtesting equipment. (f) Schematic of TE and TM polarization. (g) Measured reflectivity of DSTEAM in the 18–40 GHz range. (h) Measured absorption properties of DSTEAM under TE polarization at different incident angles in the 2.6–18 GHz range. (i) Measured absorption properties of DSTEAM under TM polarization at different incident angles in the 2.6–18 GHz range. (j) Measured absorption properties of DSTEAM under TE polarization at different incident angles in the 18–40 GHz range. (k) Measured absorption properties of DSTEAM under TM polarization at different incident angles in the 18–40 GHz range. (lq) Three‐dimensional bistatic RCS simulation results of a PEC plate vs. a plate coated with DSTEAM at 4.5 GHz (l and m), 9 GHz (n and o), and 16 GHz (p and q). (rt) Comparison of the RCS between a PEC plate and a plate coated with DSTEAM at 4.5 GHz (r), 9 GHz (s), and 16 GHz (t).

Through machine learning‐driven parameter optimization for DSTEAM, the optimal parameters obtained are: A = 26°, W = 2 mm, H = 17 mm, and L = 58 mm. Based on these optimized structural parameters, a DSTEAM sample with dimensions of 180 × 180 mm was fabricated (Figure 7a). A comparison between the simulated reflectivity and the experimentally measured reflectivity is presented in Figure 7b. It can be observed that the variation trend of the simulated reflectivity is largely consistent with that of the experimentally measured one. The existing discrepancies may be attributed to the difficulty in achieving complete consistency between the structural parameters in practical fabrication and those used in the simulation.

The absorption characteristics under different incidence angles are illustrated in Figure 7h,j for TE polarization and Figure 7i,k for TM polarization. The material exhibits excellent polarization‐insensitive properties, maintaining over 90% absorption efficiency even at an incidence angle of 45°. It is noteworthy that although the simulation was focused on the 2.6–18 GHz frequency band, experimental validation extended the frequency range up to 40 GHz. The results demonstrate continuous effective absorption (reflection loss ≤ –10 dB) across 2.6–40 GHz, achieving an ultra‐wide EAB of 37.4 GHz (Figure 7b,g).

**FIGURE 8:**
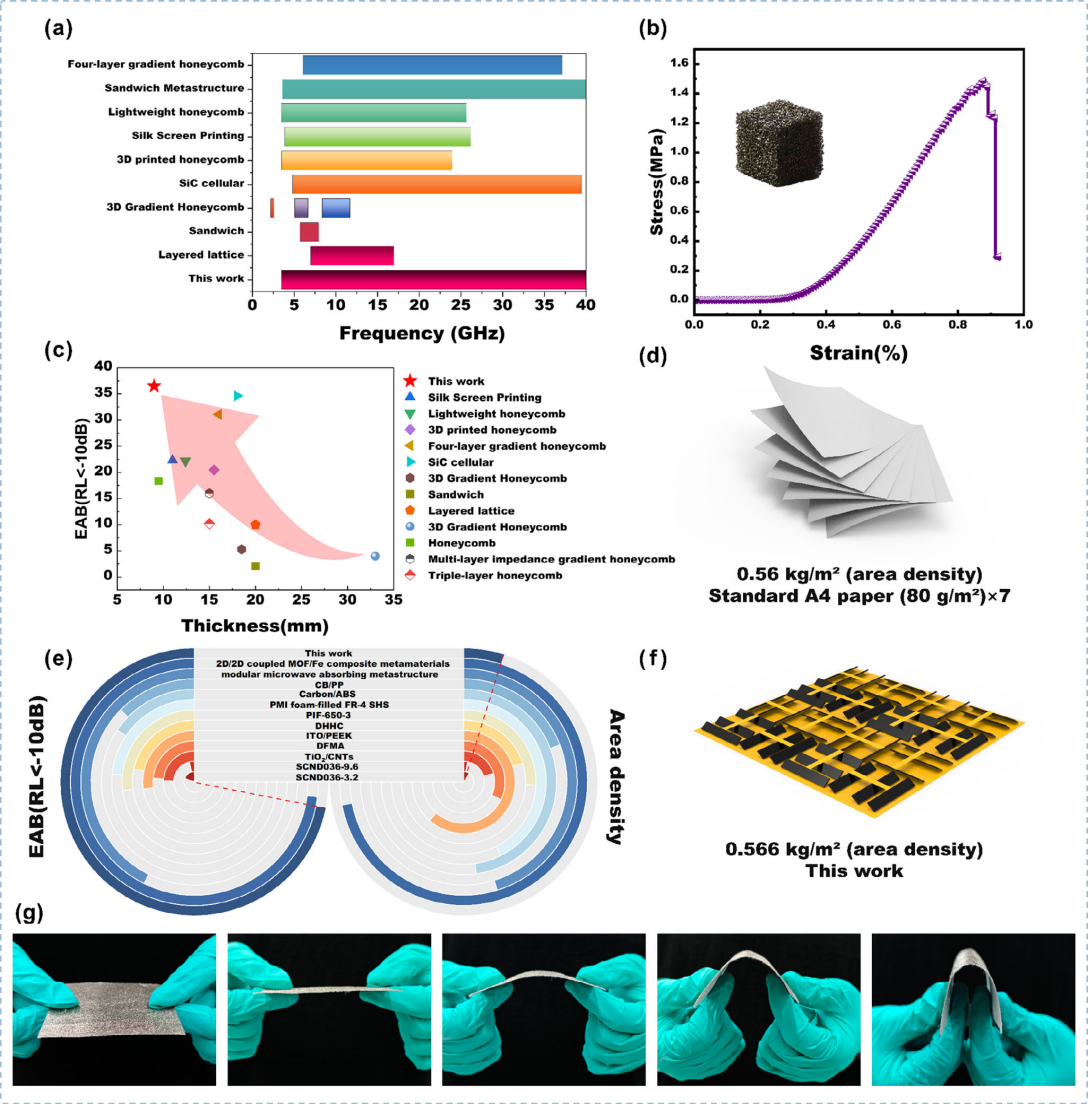
(a) Bandwidth performance comparison between DSTEAM and other electromagnetic wave‐absorbing materials. (b) Compressive test of the porous carbon material. (c) Thickness performance comparison between DSTEAM and other electromagnetic wave‐absorbing materials. (d) Areal density of seven sheets of standard A4 paper. (e) Areal density of DSTEAM. (f) Areal density comparison between DSTEAM and other electromagnetic wave‐absorbing materials. (g) Flexibility test of the magnetic material.

As shown in Figure 7c, this depicts the field testing scenario of the material in a natural environment. Compared to standardized testing under ideal laboratory conditions, field environments are often accompanied by complex factors such as multipath interference, weather variations, and ground reflections. Therefore, field tests can more realistically reflect the material's actual performance in engineering applications. To ensure stable and reliable outdoor testing, the measurements were conducted at an open site. The sample was placed on a platform with a low dielectric constant, maintaining sufficient height above the ground. The weather conditions during testing were as follows: clear skies, temperature 23°C, relative humidity 69%, wind speed 3 m/s, and atmospheric pressure 103.5 kPa. Twenty independent measurements were performed for each frequency band, and the final results are presented with accompanying error bands. The test results indicate that the material maintains an effective absorption bandwidth of 2.6–40 GHz even in the field environment (Figure 7b,g). This not only further validates the material's excellent electromagnetic wave absorption performance but also fully demonstrates its promising application potential.

Radar cross section (RCS) is a key metric for evaluating the radar stealth potential of materials. In this study, we further conducted RCS simulation analysis [36, 37, 38]. Building upon the excellent reflection loss performance obtained earlier, a plate model coated with the designed absorbing material was constructed. Three characteristic frequencies near the reflection loss resonance peaks—4.5, 9, and 16 GHz—were selected to simulate the electromagnetic scattering characteristics under plane wave incidence. Figure 7(l–q) compare the three‐dimensional bistatic RCS simulation results between the plate coated with the absorbing material and an identical‐sized perfect electric conductor (PEC) plate at different frequencies, showing a reduction in scattering intensity and a significant decrease in RCS. To further quantify the stealth performance, Figure 7(r–t) present two‐dimensional comparison curves of RCS values vs. observation angle. At all three characteristic frequencies, the plate coated with the absorbing material achieves notable RCS reduction compared to the PEC plate. This result directly demonstrates that the material can effectively absorb electromagnetic waves, thereby substantially lowering the probability of target detection. This simulation study bridges the waveabsorbing performance of the DSTEAM material with macroscopic engineering application metrics, providing crucial theoretical support and quantitative reference for its potential application in radar stealth technology.

### 2.3 Performance Advantages of DSTEAM

As shown in Figure 8, the optimized DSTEAM demonstrates outstanding performance across multiple key metrics. Its effective absorption bandwidth (EAB) covers the full 2.6–40 GHz frequency range (Figure 8a), exhibiting broader coverage compared to many absorber materials reported in the literature. Simultaneously, the material maintains a relatively low overall thickness even under broadband absorption conditions (Figure 8c) [18, 39, 40, 41, 42, 43, 44, 45, 46, 47, 48, 49, 50]. Benefiting from its porous skeleton structure, the material achieves an ultra‐low areal density of only 0.566 kg/m^2^ (Figure 8f), which is comparable to seven sheets of standard A4 paper (Figure 8d) and significantly lower than that of similar materials (Figure 8e) [41, 51, 52, 53, 54, 55, 56, 57, 58, 59, 60, 61]. Mechanical tests indicate that the porous carbon material possesses good compressive strength (Figure 8b), while the magnetic component also exhibits certain flexibility (Figure 8g). These properties collectively indicate that DSTEAM offers well‐balanced, comprehensive performance in terms of broadband absorption, lightweight design, and structural adaptability. This makes DSTEAM a promising candidate for weight‐ and space‐sensitive application fields, such as aerospace and mobile communication platforms.

### 2.4 Tunability Analysis and Mechanism Investigation

**FIGURE 9:**
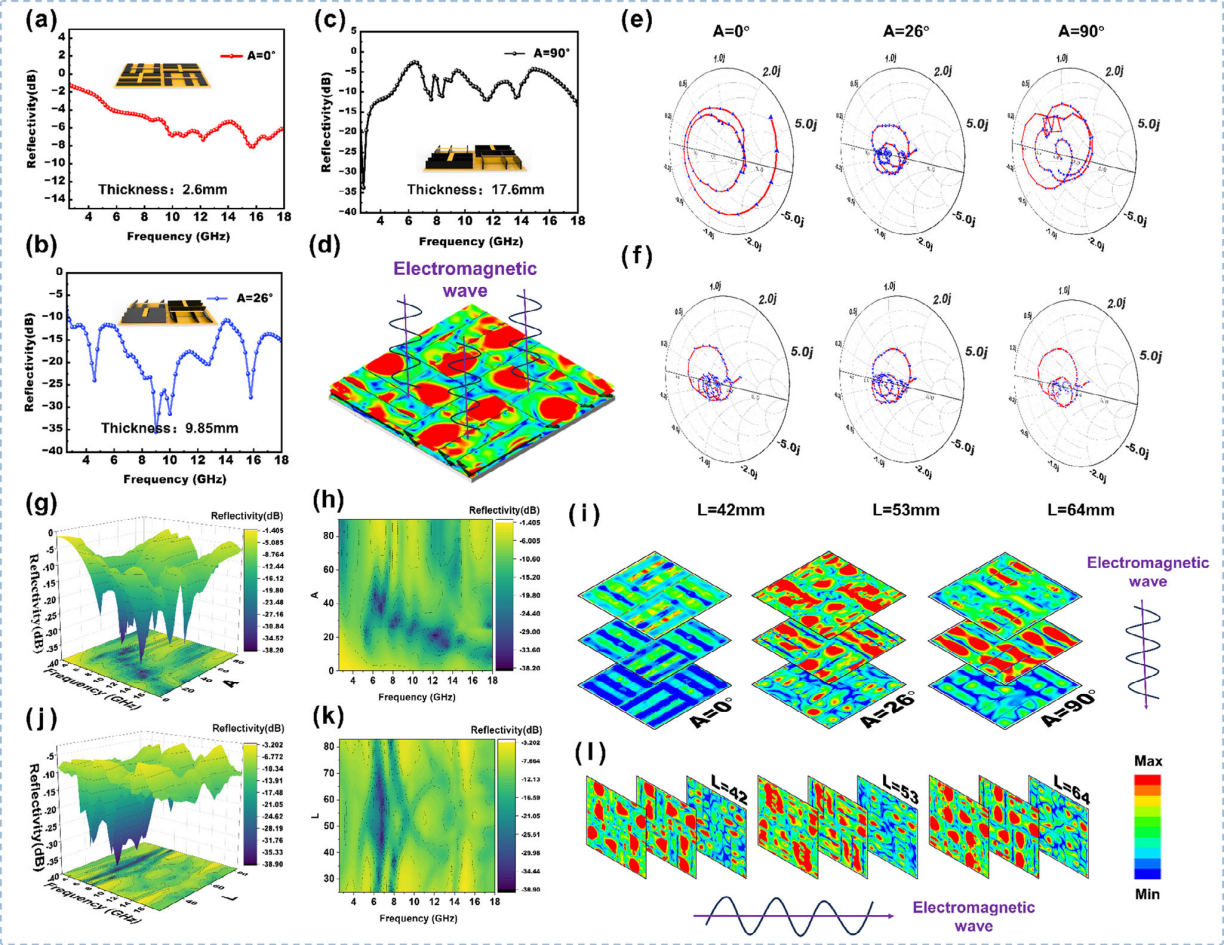
(a–c) Tunability demonstration of DSTEAM (0°, 26°, 90°) and comparison of reflectivity at different tilt angles. (d) Schematic of gradient electric field slice analysis. (e) Comparison of Smith charts at different tilt angles (0°, 26°, 90°). (f) Comparison of Smith charts at different inter‐slat distances (42, 53, 64 mm). (g and h) 3D reflectivity map and contour plot showing the variation of DSTEAM reflectivity with tilt angle. (i) Gradient electric field slice analysis at different tilt angles. (j and k) 3D reflectivity map and contour plot showing the variation with inter‐slat distance parameter. (l) Gradient electric field slice analysis at different inter‐slat distances.

The DSTEAM designed in this study features an adjustable inclination angle (Figure 9a–c). By mechanically tuning the tilt angle of the slat units (0°–90°), the electromagnetic response can be dynamically optimized. Experimental results demonstrate that at an inclination of 90°, the material achieves a reflection loss peak of –34.5 dB in the low‐frequency band. When the tilt angle is adjusted to 26°, the effective EAB reaches 15.4 GHz within the 2.6–18 GHz range. This angle‐dependent tunability enables the material to rapidly adapt to different application scenarios, providing intelligent wave‐absorption functionality with frequency‐tunable and performance‐controllable characteristics. Both experimental and simulation results reveal that the laterally heterogeneous structure creates a non‐uniform distribution of electromagnetic energy in the longitudinal direction, ultimately forming a broadband energy dissipation gradient field perpendicular to the surface. This two‐dimensional horizontal gradient discrete design constructs a gradient‐like structure within the lateral space, which enhances impedance matching and improves wave absorption performance without increasing the overall thickness.

To elucidate the underlying mechanism of the enhanced impedance matching and broadband absorption of DSTEAM, simulations were conducted using HFSS for a configuration with a fixed thickness of 2 mm and height of 17 mm under various inclination angles and inter‐slit distances. Comparative Smith chart analysis (Figure 9e) indicates that as the tilt angle increases, the equivalent thickness decreases, leading to improved impedance matching. This allows more incident electromagnetic waves to penetrate the material, significantly enhancing dissipation capability. Furthermore, adjustments in the inter‐slit distance (Figure 9f) alter the material density distribution, resulting in modulated impedance matching. This design strategy establishes a horizontally graded and discretized impedance matching layer along the lateral dimension.

To quantitatively reveal the variation of the gradient impedance distribution with respect to the slat angle and gap spacing, this study further investigates the structural parameter sweep and electric field simulation analysis of the system. Figure 9d shows a schematic diagram of the gradient electric field slice analysis. Based on the control variable method (with H = 17 mm and W = 2 mm fixed), the 3D reflectivity maps and contour plots in Figure 9 quantitatively illustrate the variation of absorption performance with tilt angle (Figure 9g,h) and inter‐slat distance (Figure 9j,k). As the tilt angle increases, the absorption resonance shifts from low to high frequencies, while the low‐frequency reflectivity shows a trend of first decreasing and then varying. When altering the inter‐slat distance, the low‐frequency and high‐frequency absorption peaks exhibit a symmetrical movement towards each other. These sweep results clarify, from a macroscopic frequency response perspective, the regulatory effect of structural parameters on the absorption peak positions.

To intuitively quantify the evolution of the impedance matching gradient, Figure 9i,l display the electric field intensity distribution along the material depth for different configurations. By comparing the electric field attenuation profiles across planes at different heights, it is found that when the tilt angle is adjusted within a specific range, the electric field energy can effectively propagate to the bottom of the material (Figure 9i), indicating the formation of a favorable longitudinal impedance gradient. Modifying the inter‐slat distance can significantly reshape the transverse distribution of the electric field within the material (Figure 9l). Working synergistically with angle adjustment, this enables the quantitative control of the equivalent impedance gradient in two‐dimensional space. The aforementioned electric field analysis directly visualizes the attenuation gradient of electromagnetic waves within the structure, demonstrating that adjusting both the tilt angle and the inter‐slat distance can actively construct and optimize the gradient impedance matching while reducing the overall material thickness.

**FIGURE 10:**
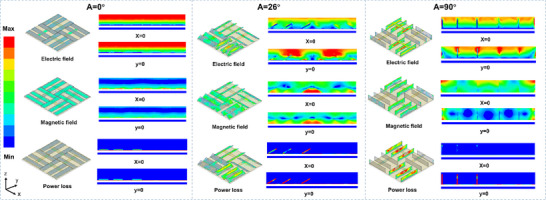
Distributions of the electric field, magnetic field, and power loss density for DSTEAM at inclination angles of 0°, 26°, and 90°.

As shown in Figure 10, adjusting the inclination angle of the DSTEAM structure effectively modulates the distribution of energy along the longitudinal gradient. Experimental results indicate that as the tilt angle decreases from 90° to 26°, the energy distribution within the material transitions significantly from a dispersed state to a concentrated one, with a markedly increased degree of localization. Concurrently with this transition, the magnetic field evolves from a discrete distribution on the material surface into a distinctly localized state, while the electric field concentrates toward the center of the bottom layer and interacts with the underlying magnetic material, resulting in interfacial polarization. This tilt angle‐induced energy localization substantially enhances the electromagnetic loss capability of the material. This tuning strategy drives the transition of the material from a non‐localized (low‐loss) to a localized (high‐loss) state, thereby improving electromagnetic dissipation performance while simultaneously reducing material thickness.

**FIGURE 11:**
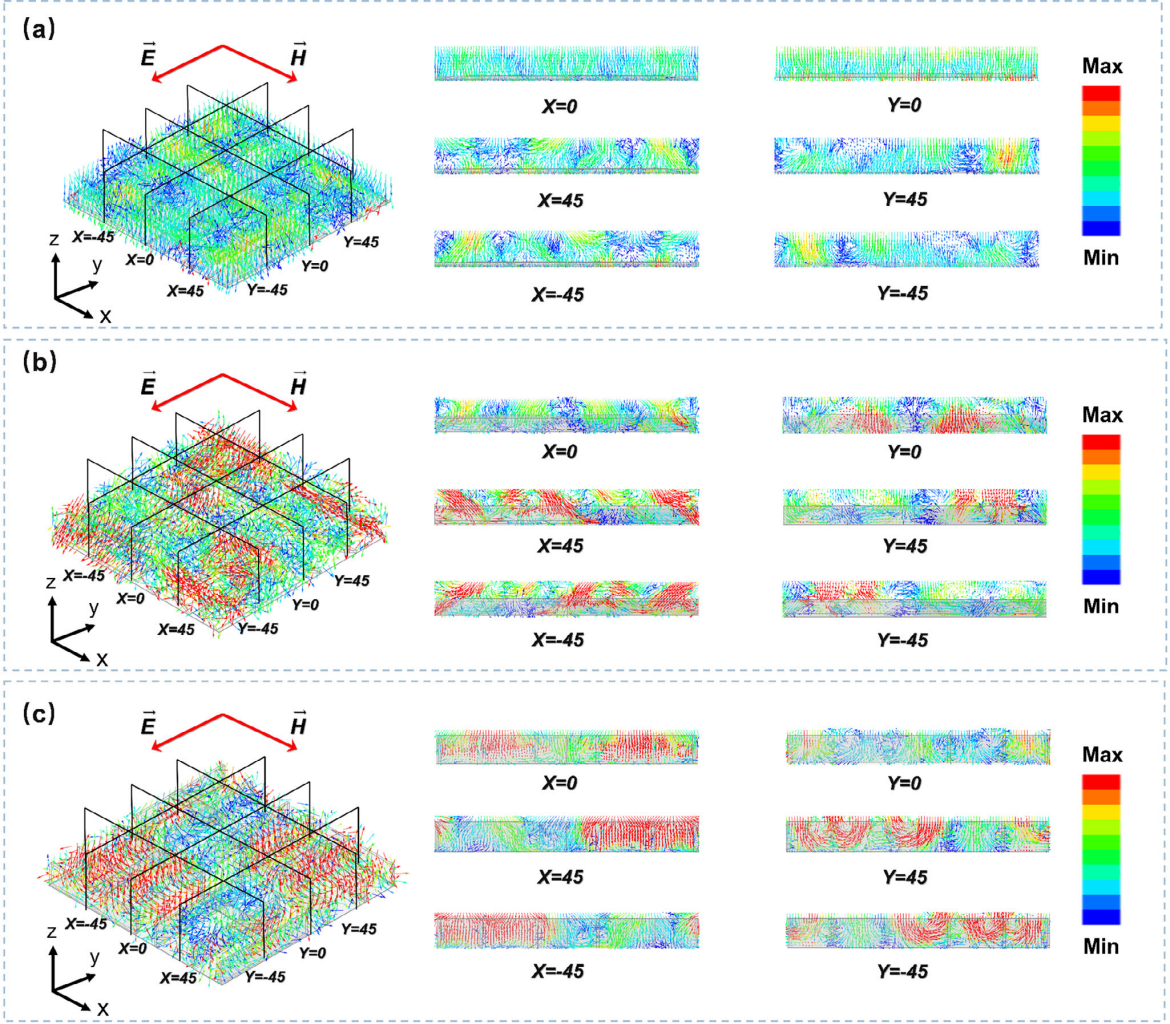
Analysis of the Poynting vector field for DSTEAM at inclination angles of (a) 0°, (b) 26°, and (c) 90°.

The distribution of the Poynting vector field (Figure 11) demonstrates that the introduction of an inclination angle in the porous carbon sheets significantly enhances the multiple scattering effects of electromagnetic waves within the material. This geometric modulation strategy prolongs the propagation path of the waves, and the resultant loss from extended paths and multiple reflections increases the total energy dissipation within the material, thereby improving the overall energy dissipation efficiency. The study further reveals that the wave‐delay effect induced by the tilted structure effectively extends the propagation path of the electromagnetic waves, markedly increasing energy attenuation inside the material. This provides an effective structural design strategy for optimizing broadband wave‐absorption performance.

**FIGURE 12:**
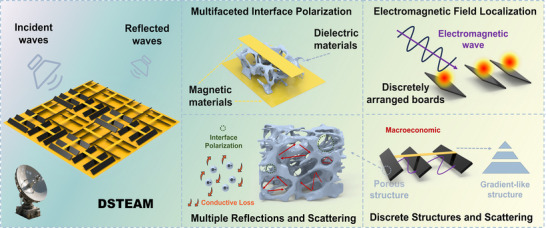
Schematic diagram of the wave‐absorption mechanism of DSTEAM.

The performance advantages of DSTEAM primarily originate from the intrinsic absorption of porous carbon, magneto‐electric coupling modulation, and the gradient‐interface multiple scattering, as well as synergistic local field enhancement induced by the blinds‐inspired discrete slat structure. As illustrated in Figure 12, the absorption mechanisms can be summarized as follows:

The magnetic and dielectric materials help improve impedance matching with incident microwaves and facilitate the formation of multiple heterogeneous interfaces, leading to significant interfacial polarization effects. The blinds‐inspired discrete slat structure effectively promotes electromagnetic field localization, enhancing the concentration and dissipation of electromagnetic energy. Furthermore, this design not only strengthens multiple reflections and scattering at gradient interfaces but also constructs a vertically graded impedance‐like structure through horizontally varying density distribution, thereby promoting wave penetration into the material.

The porous carbon skeleton provides a continuous conductive network, facilitating electron migration and enhancing conductive loss. Meanwhile, its abundant porous architecture prolongs the propagation and dissipation path of electromagnetic waves and introduces numerous solid–gas interfaces, which are conducive to interfacial polarization and multiple scattering. The synergistic interaction of these mechanisms collectively enhances the wave‐absorption performance of the material, achieving multi‐scale structural design that enables multi‐level transmission and loss mechanisms.

## 3 Conclusions

Inspired by the structure of window blinds, this study developed a Discrete Slat Tunable DSTEAM by integrating a magneto‐electric coupling mechanism with an artificial intelligence‐assisted data‐driven approach. Utilizing a neural network surrogate model (R^2^ > 0.97) coupled with a genetic algorithm, an intelligent inverse design of structural parameters was achieved, significantly shortening the traditional trial‐and‐error design cycle (with an approximately 50‐fold improvement in efficiency). The optimized DSTEAM exhibits an effective absorption bandwidth (reflection loss ≤ –10 dB) covering the full frequency range of 2.6–40 GHz, while maintaining an ultralow thickness of 9.85 mm and an areal density of 0.566 kg/m^2^. The superior performance of DSTEAM originates from gradient‐induced multiple scattering at discrete sheet interfaces, a synergistic enhancement of localized field strength, and a magneto‐electric coupling modulation mechanism. These features collectively optimize impedance matching and enhance loss capability while reducing material thickness. Moreover, the absorber maintains over 90% wave‐absorption efficiency even at a wide incidence angle of up to 45°. The proposed design strategy, which integrates bio‐inspired configuration and intelligent optimization, provides a theoretical framework and technical pathway for the development of next‐generation lightweight and broadband absorbers. It demonstrates promising application potential in intelligent stealth skins and adaptive electromagnetic protection.

## 4 Experimental Section

### 4.1 Material Preparation (Porous Carbon and Magnetic Material)

#### 4.1.1 Porous Carbon

Using polyurethane (PU) sponge foam as a template, the sponge was cut into rectangular blocks measuring 200 mm × 200 mm × 20 mm. After ultrasonic cleaning with ethanol and deionized water, followed by drying, the sponge was immersed in a slurry composed of phenolic resin and a curing agent in a mass ratio of 10:0.05. The sponge was squeezed to facilitate infiltration, and excess slurry was removed by centrifugation. It was then cured at 80°C for 20 min, with the impregnation and curing process repeated several times. The treated sponge was pyrolyzed in an Ar atmosphere at a heating rate of 4°C/min up to 800°C and held for 2 h to obtain the porous carbon.

#### 4.1.2 Magnetic Material

Polyvinyl alcohol (PVA) particles were mixed with deionized water at a mass ratio of 1:9, heated to 90°C, and stirred for approximately 1 h until fully dissolved to obtain a 10 wt.% PVA solution. Fe‐Si‐Al powder was ball‐milled with the PVA solution at a mass ratio of 1:10. The resulting slurry was coated onto wet‐laid paper using a doctor blade method and dried to form a flexible magnetic composite film with a thickness of 0.3 mm.

### 4.2 Characterization

The morphology of the samples was characterized using scanning electron microscopy (SEM, SEM5000) and transmission electron microscopy (TEM, FEI Talos F200X). Phase identification was performed using high‐throughput X‐ray diffraction (XRD, D8 Advance). Raman spectroscopy (LabRam Odyssey, Horiba, Japan) was employed to obtain Raman spectra for identifying characteristic peaks and analyzing the composition and chemical structure of the samples. The surface elemental composition and valence states were analyzed by X‐ray photoelectron spectroscopy (XPS, Axis Supra+). Microstructural features were observed via three‐dimensional reconstruction using high‐resolution 3D X‐ray microscopy (3D‐XRT, Xradia 620 Versa). Chemical bonds and functional groups were analyzed by Fourier‐transform infrared spectroscopy (FTIR, IS50). Pore size distribution, porosity, and related parameters were quantitatively determined using a mercury intrusion porosimeter (AutoPore V960, Micromeritics Instrument Corporation). The compressive strength of the material was tested with a universal testing machine (WDW‐50).

### 4.3 Electromagnetic Property Measurement and Simulation

The electromagnetic parameters of the samples were measured using a vector network analyzer (PNA, Keysight N5225B). The complex permittivity of the porous carbon was determined via the waveguide method over the frequency range of 2.6–18 GHz. The waveguide dimensions were as follows: 86.4 mm × 43.2 mm for 2.6–3.95 GHz, 47.5 mm × 22.1 mm for 3.95–5.85 GHz, 34.8 mm × 15.7 mm for 5.85–8.2 GHz, 22.8 mm × 10.1 mm for 8.2–12.4 GHz, and 15.7 mm × 7.8 mm for 12.4–18 GHz. The thickness of the waveguide samples was between 4 and 6 mm. The electromagnetic parameters of the FeSiAl material were measured using the coaxial method over the frequency range of 2.6–18 GHz. The coaxial toroidal sample had an inner diameter of 3.04 mm, an outer diameter of 7.00 mm, and a thickness of 2 mm. The reflectivity samples had dimensions of 180 mm × 180 mm. Design and simulation work were performed using the HFSS software.

### 4.4 Machine Learning Surrogate Model Generation and Optimization

#### 4.4.1 Construction and Training of the Surrogate Model

The design of the absorber was parameterized by defining four key geometric parameters: length, width, thickness, and tilt angle. To comprehensively cover the design space, 3000 parameter combinations were generated using the Latin Hypercube Sampling method, ensuring uniformity and representativeness across the parameter space. For each set of geometric parameters, the absorption ratio across the frequency band of 2.6–18 GHz, with a step size of 0.1 GHz, was calculated using HFSS simulations. Thus, a dataset for training the surrogate model was constructed, comprising 3000 samples. Each sample has the four‐dimensional geometric parameters as input features and the corresponding 155‐dimensional absorption ratio vector as the output label.

A fully connected neural network was built as the surrogate model using Python 3.8 and the PyTorch framework. The input layer of the network corresponds to the 4 geometric parameters, and the output layer consists of 155 neurons with a linear activation function to directly regress the absorption ratio curve across the entire frequency band. The hidden section of the network consists of 3 layers, each containing 128 neurons, and employs the ReLU activation function to introduce nonlinearity. The model was trained using the Adam optimizer with the Mean Squared Error (MSE) as the loss function. To prevent overfitting, the dataset was randomly split into training, validation, and test sets in a 7:2:1 ratio, and the early stopping technique was applied. Ultimately, after 200 training epochs, the model achieved satisfactory accuracy on the test set. Its reliability as a high‐precision surrogate model is confirmed by the average coefficient of determination (R^2^) between its predictions and the simulation results.

#### 4.4.2 Optimization Design Based on the Genetic Algorithm

Following the acquisition of the high‐precision surrogate model, a Genetic Algorithm was employed to optimize the geometric parameters. The objective was to maximize the number of frequency points (i.e., the fitness value, with a maximum possible value of 155) within the 2.6–18 GHz band where the absorption ratio exceeds 90%. The algorithm started by randomly generating an initial population of 100 candidate structures. It then proceeded iteratively: the trained neural network surrogate model predicted the absorption curve for each individual and calculated its fitness. A new generation was created through selection, crossover (using simulated binary crossover), and mutation (using polynomial mutation with a probability of 10%) operations, while an elitism strategy was incorporated to prevent the loss of high‐fitness individuals. This optimization cycle continued until either a maximum of 20 generations was reached or the optimal fitness stabilized with no further improvement, thereby identifying the optimal absorber.

## Funding

The National Natural Science Foundation of China (Grant No. 12305069), the Program of the Education Department of Liaoning Province (Grant No. JYTMS20231695)

## Conflicts of Interest

The authors declare no conflicts of interest.

## Data Availability

The data that support the findings of this study are available on request from the corresponding author.
